# Prevalence of PTSD in Survivors of Stroke and Transient Ischemic Attack: A Meta-Analytic Review

**DOI:** 10.1371/journal.pone.0066435

**Published:** 2013-06-19

**Authors:** Donald Edmondson, Safiya Richardson, Jennifer K. Fausett, Louise Falzon, Virginia J. Howard, Ian M. Kronish

**Affiliations:** 1 Center for Behavioral Cardiovascular Health, Columbia University Medical Center, New York, New York, United States of America; 2 College of Physicians and Surgeons, Columbia University Medical Center, New York, New York, United States of America; 3 Department of Psychiatry, University of Arkansas for Medical Sciences, Little Rock, Arkansas, United States of America; 4 Department of Epidemiology, School of Public Health, University of Alabama at Birmingham, Birmingham, Alabama, United States of America; University of Münster, Germany

## Abstract

**Background and Purpose:**

Posttraumatic stress disorder (PTSD) is common in survivors of acute life-threatening illness, but little is known about the burden of PTSD in survivors of stroke and transient ischemic attack (TIA). This study estimated the prevalence of stroke or TIA-induced posttraumatic stress disorder (PTSD) using systematic review and meta-analysis.

**Methods:**

Potentially relevant peer-reviewed journal articles were identified by searching the Ovid MEDLINE, PsycINFO, PILOTS Database, The Cochrane Library and Scopus from inception to January 2013; all searches were conducted on January 31, 2013. Observational cohort studies that assessed PTSD with specific reference to a stroke or TIA that occurred at least 1 month prior to the PTSD assessment were included. PTSD rates and characteristics of the study and sample were abstracted from all included studies. The coding of all articles included demographics, sample size, study country, and method and timing of PTSD assessment.

**Results:**

Nine studies (N = 1,138) met our inclusion criteria. PTSD rates varied significantly across studies by timing of PTSD assessment (i.e., within 1 year of stroke/TIA versus greater than 1 year post-stroke/TIA; 55% of heterogeneity explained; *Q*_1_ = 10.30; *P* = .001). Using a random effects model, the estimated rate of PTSD following stroke or TIA was 23% (95% CI, 16%–33%) within 1 year of the stroke or TIA and 11% (95% CI, 8%–14%) after 1 year.

**Conclusions:**

Although PTSD is commonly thought to be triggered by external events such as combat or sexual assault, these results suggest that 1 in 4 stroke or TIA survivors develop significant PTSD symptoms due to the stroke or TIA. Screening for PTSD in a large population-based prospective cohort study with cardiovascular outcome assessments is needed to yield definitive prevalence, and determine whether stroke or TIA-induced PTSD is a risk factor for subsequent cardiovascular events or mortality.

## Introduction

Posttraumatic stress disorder (PTSD) is an anxiety disorder initiated by exposure to a traumatic event and is characterized by symptoms of re-experiencing, avoidance of reminders of the event, persistent negative mood and cognition, and physiological hyperarousal that persist for at least 1 month after the event. Observational evidence suggests that PTSD is related to increased risk of incident cardiovascular disease [1] [2]. A recent meta-analysis further suggests that PTSD *triggered* by cardiovascular events - specifically, acute coronary syndrome (ACS) - is associated with a doubling of risk for recurrent cardiac events and mortality [3] [4].

Until recently, only a few studies assessed PTSD due to stroke, another potentially traumatic acute cardiovascular event. Prevalence estimates in these studies has varied from 3–37% [5] [6] [7] [8] [9] [10] [11] [12]. A more recent study, the largest on this topic to date, found that 18% of 535 stroke or TIA survivors reported clinically significant PTSD symptoms. While the association between stroke or TIA-induced PTSD and risk for recurrent stroke or other cardiovascular events was not examined, participants in this study were nearly three times more likely than those without PTSD symptoms to report medication nonadherence [13]. These data suggest that, similar to ACS-induced PTSD, stroke or TIA-induced PTSD may increase risk for recurrent stroke and other cardiovascular events. This meta-analytic review of cross-sectional studies was designed to estimate the overall prevalence of PTSD due to stroke or TIA.

## Methods

### Search Strategy and Selection Criteria

We sought to identify all studies that reported a valid prevalence estimate of PTSD due to stroke or TIA. Included studies must have been observational cohorts and must have assessed PTSD with specific reference to a stroke or TIA that occurred at least 1 month prior to the PTSD assessment. Further, studies must have used a self-report PTSD screening instrument or clinical interview designed or specifically altered to query about only *stroke or TIA-induced* PTSD. For the one study that reported multiple PTSD assessments, we abstracted only the earliest estimate (at 1 month post-stroke) [10].

Potentially relevant peer-reviewed journal articles published in English were identified by a search of the biomedical electronic databases Ovid MEDLINE, PsycINFO, PILOTS Database, The Cochrane Library and Scopus conducted by an information specialist (L.F.) with expertise in behavioral medicine. Databases were searched from inception to January 2013; all searches were conducted on January 31, 2013. All relevant subject headings and free-text terms were used to represent PTSD and stroke or TIA and the sets of terms were combined with AND. Terms for MEDLINE included: exp Stress Disorders, Traumatic/OR ptsd.tw. OR (post-traumatic OR (post adj traumatic)).tw or posttraumatic.tw. AND exp stroke/OR Stroke$.tw OR cerebrovascular.tw. OR ((brain or vascular or lacunar or venous or cerebral or isch?emic) adj2 (accident$ or infarct$ or event$ or attack$)).tw. Search terms were adapted for the other databases. Additional records were identified by scanning the reference lists of relevant studies and reviews and by employing the Related Articles feature in PubMed/Medline and the Cited Reference Search in Scopus. To determine the studies to be assessed further, two authors (S.R., D.E.) independently read the title, or title and abstract of every record retrieved. Opinion differences were resolved by consensus. All potentially relevant articles were investigated as full text.

### Database Construction and Coding

We abstracted PTSD rates and characteristics of the study and sample from all included studies (**Table 1**). The coding of all articles included demographic information, sample size, study country, and method and timing of PTSD assessment. We also evaluated the quality of each study based on the Methodological Evaluation of Observational Research (MORE)- Observational Studies of Incidence or Prevalence of Chronic Diseases developed by the Agency for Healthcare Research and Quality (AHRQ) [14]. One study’s prevalence estimate, ^7^ and another’s demographic characteristics were uncertain [9], so authors were contacted to obtain this information. We converted one study’s prevalence estimate to make it consistent with the method used by the other studies that estimated prevalence using a screening questionnaire [13]. All studies that were included used either diagnostic interviews or well-validated self-report screening questionnaires that assessed PTSD *specifically due to stroke or TIA*.

**Table 1:** Characteristics of Studies on the Prevalence of Stroke-Induced PTSD.

Source, y	PTSD Prevalence,%	PTSDMeasure	Clinical Interview, Y/N	Time Post- Stroke, mo	Study Location	N	Male, %	Mean Age, y
Bruggiman et al., 2006	31	IES	N	1–12	Switzerland	49	67	51
Field, 2008	37	PDS	N	3	United Kingdom	70	53	71
Kronish, 2012	12	PCL	N	1–60	United States	535	41	63
Letamendia, 2012	4	PCL	N	1	France	27	63	64
Merriman, 2007	31	PDS	N	1–12	United Kingdom	102	56	74
Noble, 2008	37	PDS	N	3	United Kingdom	97	43	52
Sagen, 2009	3	SCID	Y	4	Norway	104	59	65
Sembi, 1998	10	CAPS	Y	1–18	United Kingdom	61	NR	66
Wang, 2011	30	PDS	N	1	United Kingdom	90	48	75

Abbreviations: CAPS, Clinician Administered PTSD Scale ^20^; IES, Impact of Events Scale ^21^; NR, not reported; PCL, PTSD Checklist ^22^; PDS, Posttraumatic Stress Diagnostic Scale ^23^; PTSD, posttraumatic stress disorder; SCID, Structured Clinical Interview for *DSM-IV* ^24^; TIA, transient ischemic attack; NR, not reported.

### Quantitative Methods

Comprehensive Meta-analysis (version 2, BioStat Software, Engelwood, NJ) served as the statistical platform for completing all statistical tests and associated graphic results. To summarize the prevalence findings, we computed prevalence point estimates using these formulas:

Logit Event Rate = Log [Event Rate/(1 − Event Rate)].

Event Rate Standard Error = √(1/(Event Rate ·Sample Size)/(1/[(1−Event Rate) · Sample Size]),

We computed 95% confidence intervals (CIs) using this formula:

Lower Limit = Logit Event Rate − (1.96 · Logit Event Rate Standard Error).

Upper Limit = Logit Event Rate+(1.96 · Logit Event Rate Standard Error).

Results are presented after back-transformation.

There was statistically significant heterogeneity in prevalence estimates, so random-effects models were used. In addition, we used sensitivity analyses to assess evidence of moderator effects for the prevalence results across two methodological factors: (1) method and (2) timing of PTSD assessment. Finally, we produced a forest plot and calculated both the classic and Orwin’s fail-safe N to address the issue of publication bias.

## Results

### Study Population

**Figure 1:**
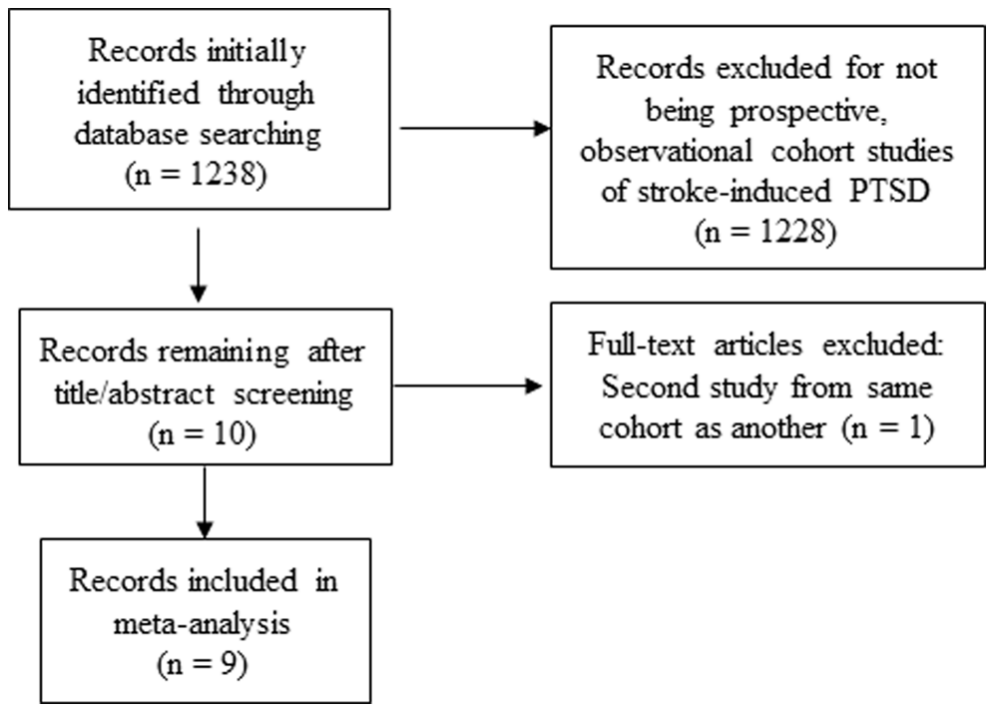
Search strategy flowchart.

Our search identified 1238 articles, and 2 coders agreed that 10 articles required full reading. Of those, 9 met our criteria for inclusion yielding a total of 1,138 participants (**Figure 1**). All participants were enrolled from either the US or Europe. The mean age of participants was 64.5 and 47.5% were men.

### PTSD Assessment

The timing of PTSD assessments ranged from 1 month to 60 months after the stroke or TIA. PTSD was assessed by clinical interview in 2 studies and using a screening questionnaire in the remaining 7 studies. Table 1 shows the screening questionnaires used in each study. While none of the scales have been validated in stroke or TIA survivors, similar screening questionnaires to those used in these studies have performed well against diagnostic interview in ACS patients [3].

### Study Quality

All included studies met our minimum criteria for quality. Study quality across the included studies was highly similar, with very good internal validity (based primarily on the use of well-validated PTSD screening questionnaires or clinical interviews) but only moderate external validity (based on nongeneral population based sampling, poor reporting of sampling bias, cross-sectional study design, poor reporting of response rates, and poor reporting of subgroup estimates across sex, race, and ethnicity groups).

### Prevalence

The prevalence estimate of stroke or TIA-induced PTSD across all studies included in the review was 13% (95% CI, 11%–16%). However, there was significant heterogeneity in prevalence rates, with reported rates ranging from 3% to 37% (*Q_9_* = 85.64; *P*<.001; *I* ^2^ = 89.49).

#### Assessment method

The PTSD prevalence estimate varied significantly by type of PTSD assessment (21% of heterogeneity explained; *Q*_1_ = 6.83; *P* = .01). The aggregate prevalence estimate in the 7 studies in which PTSD was assessed with diagnostic self-report questionnaires was 28% (95% CI, 18%–41%), compared with 6% (95% CI, 2%–18%) in the two studies in which PTSD was assessed with clinical interviews.

#### Timing of PTSD assessment

The PTSD prevalence estimate varied significantly by timing of assessment (64% of heterogeneity explained; *Q*_1_ = 10.33; *P* = .001). Of the 9 included studies, 7 assessed PTSD within 1 month to 1 year after the index stroke [6] [7] [8] [10] [11] [12]. The other 2 assessed PTSD up to 18 months [9] and 5 years (mean, 1.8 years) [13] after the index stroke. The aggregate prevalence estimate in the 6 studies in which participants were assessed within 1 year of the index stroke was 25% (95% CI, 17%–35%), compared with 12% (95% CI, 9%–15%) in those assessed in studies that allowed more time between the index stroke and PTSD assessment.

#### Final estimates

**Figure 2:**
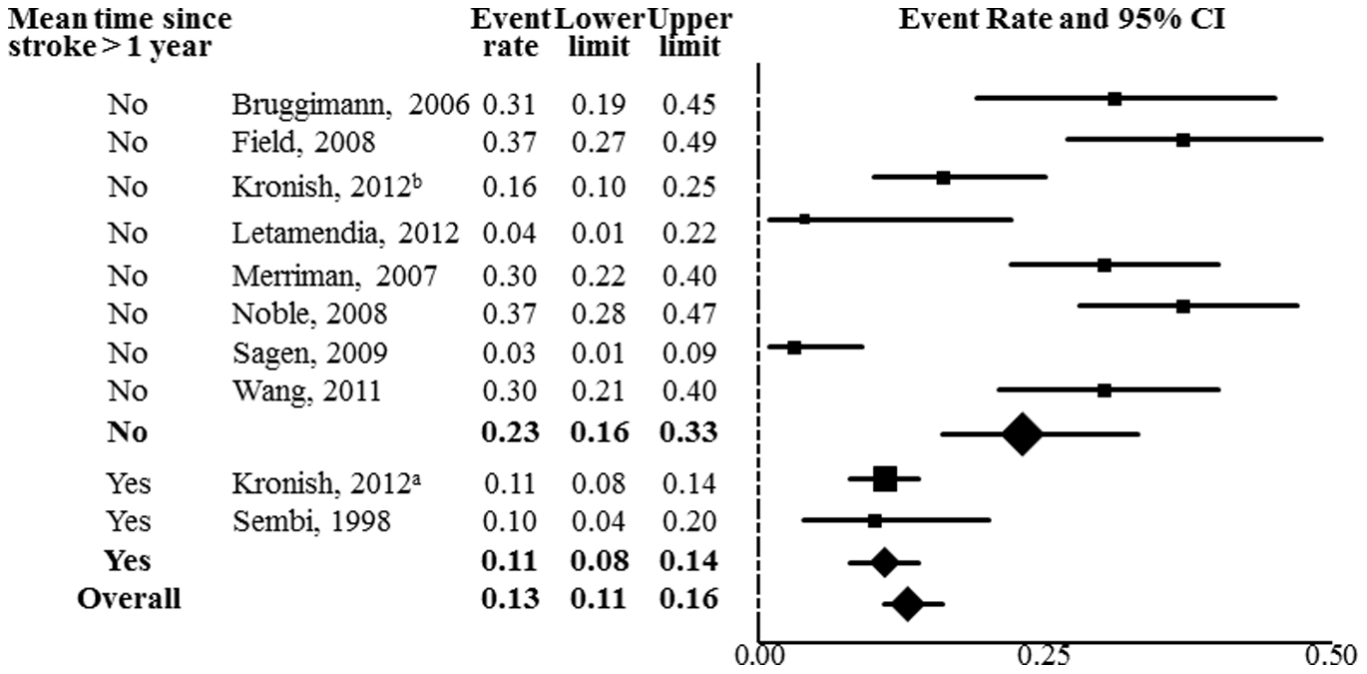
Stroke-induced PTSD prevalence estimates. Note: The area of each square is proportional to the study's weight in the meta-analysis, and each line represents the confidence interval around the estimate. Diamonds represent aggregate estimates, by PTSD assessment timing and overall, and their lateral points indicate confidence intervals for the estimates.

As substantial heterogeneity explained by timing of PTSD assessment suggested that two underlying prevalence estimates existed, we conducted another meta-analysis with the estimates from Kronish et al [13], the study with the largest sample size, divided into estimates for participants with index strokes within 12 months (prevalence = 16%) and those with index strokes greater than 12 months prior to PTSD assessment (prevalence  = 11%). **Figure 2** shows the final overall estimate of 13% (95% CI, 11%–16%) from the random effects model comprised of an aggregate estimate of 23% (95% CI, 16%–33%) for PTSD within 1 year of stroke and 11% (95% CI, 8%–14%) after 1 year. Timing of PTSD assessment continued to explain a significant proportion of the heterogeneity in prevalence estimates in the final model (55% of heterogeneity explained; *Q*_1_ = 10.30; *P* = .001).

#### Assessment of bias

**Figure 3:**
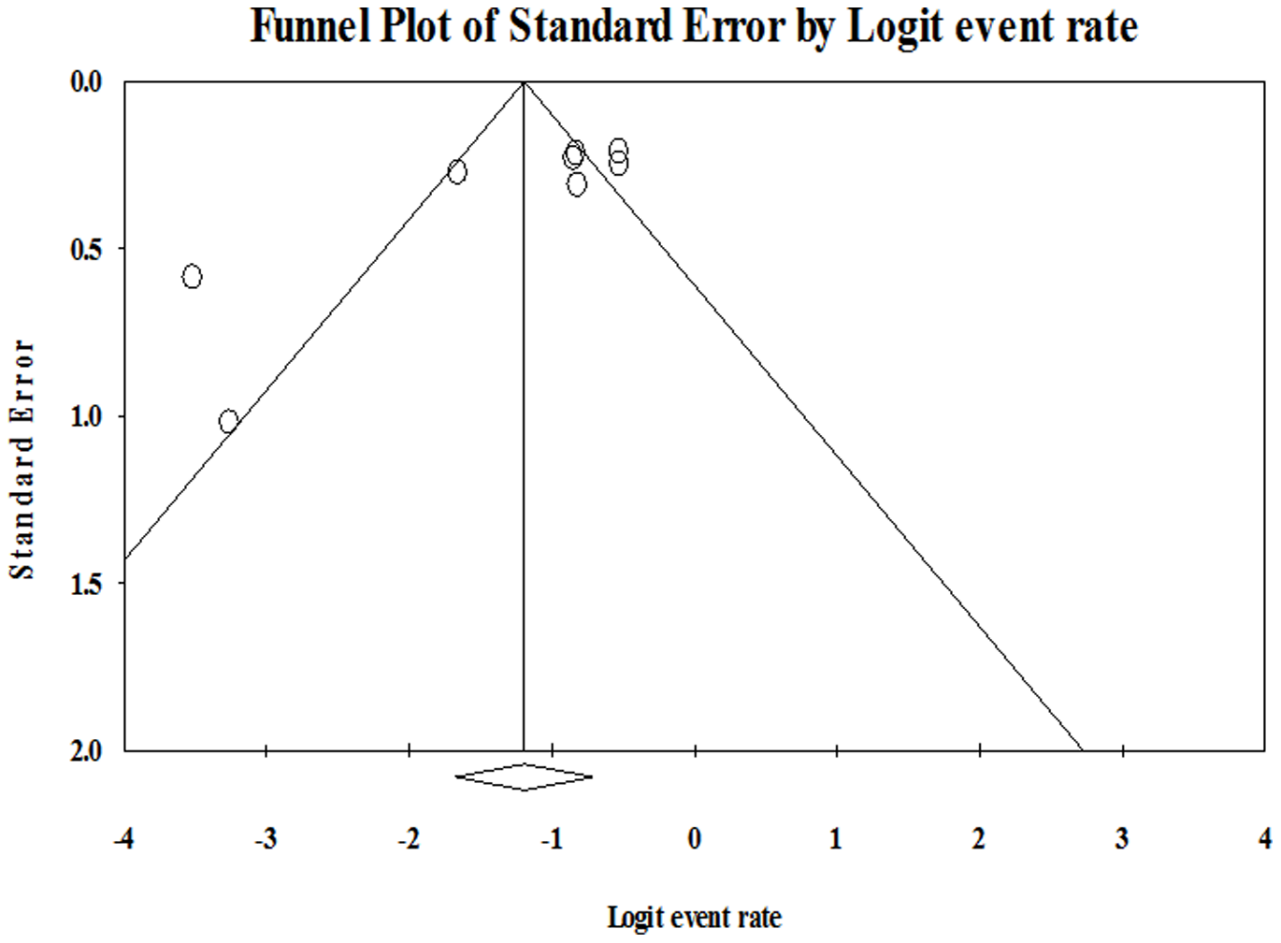
Funnel plot to assess publication bias across prevalence studies.

The forest plot given in Figure 3 does not suggest substantial publication bias. We calculated the classic fail-safe N, which suggested that 228 unpublished studies with null results would have to exist for our estimate to become statistically non-significant. However, in this study, we were more concerned with the potential influence of unpublished studies on the precision of our estimate. Orwin’s fail-safe N suggested that only 3 unpublished studies with no PTSD cases would have to exist to yield an aggregate estimate of 10% (versus the 23% we report) for the prevalence of PTSD in the first year post-stroke.

## Discussion

To our knowledge, this is the first meta-analytic review of stroke or TIA-induced PTSD. We found an overall prevalence of 13% among stroke/TIA survivors, with 23% prevalence in the first year post-stroke and 11% after the first year. Given that about 85% of the 795,000 patients who experience a stroke each year in the US [15] survive at least 30 days [16], and up to an additional 500,000 suffer from a TIA [17], these results suggest that 297,850 stroke and TIA survivors in the United States alone will develop PTSD symptoms due to the event annually. Within 90 days of the index stroke, 6% of survivors experience a recurrent stroke [18], and recurrence rates increase substantially over longer term follow up [19]. We do not yet know if stroke or TIA-induced PTSD is associated with increased risk for a recurrent cerebrovascular attack, but given the doubling of ACS recurrence risk due to ACS-induced PTSD [3] and recent research showing that PTSD is strongly associated with medication nonadherence [13], such research is sorely needed.

Important caveats to this review include the fact that only studies with relatively small sample sizes have addressed stroke-induced PTSD, and one of the studies represented half of all of the participants included in this review. Also, none of the included studies used population-based sampling, and all but one study [10] relied on cross-sectional designs (and we used only the earliest point estimate, 1 month after stroke). Further, the PTSD screening questionnaires used in many of the studies have not been validated against clinical diagnosis of PTSD in survivors of stroke/TIA, though they perform very well in other patient populations. Thus, although this meta-analysis was able to quantify more precisely the prevalence of stroke-induced PTSD symptoms, a clear need for additional research remains. It is also important to note that we were very strict in our inclusion of only studies that measured PTSD *specifically due to the stroke event,* so these estimates may represent an underestimate of the total burden of PTSD symptoms in these participants in so much as PTSD due to other types of events may have been present.

The estimated prevalence of stroke-induced PTSD was lower when PTSD was assessed by clinical interview. This may be due to the fact that the prevalence of psychiatric disorders is often higher when measured using diagnostic instruments as compared to clinical psychiatric interviews Nevertheless, research on the association between ACS-induced PTSD and ACS recurrence and mortality risk suggests that even elevated symptoms of PTSD, not clinical diagnosis, is associated with increased risk. Hence, the estimate of the prevalence of PTSD symptoms based on diagnostic instruments may still be clinically relevant [3].

### Study Limitations

Prevalence estimates were limited to patients who could participate in PTSD assessments and hence should be extrapolated to patients with severe cognitive impairment or aphasia with caution. Similarly, these results should be interpreted with awareness that the mean age of the participants was slightly low relative to the entire population of stroke survivors and PTSD is associated with younger age in ACS-induced PTSD [3], and therefore our rate estimate may not generalize to older stroke survivors. We were unable to determine whether the prevalence of PTSD differed if due to TIA or stroke, as the only study to include TIA survivors [13] did not record the proportion of participants who experienced either condition. Moreover, none of the studies reported on the severity of the stroke event in terms of the types of critical care that may have been necessitated such as intensive care unit (ICU) admission.

Only one of the studies included in this review evaluated for stroke lesion localization [6], suggesting the need to further examine whether stroke location (i.e. hemisphere, etc) or the nature of the associated deficits (i.e. paresis/paralysis, aphasia, anosagnosia) further differentiate PTSD risk. Such knowledge would allow clinicians to identify those at higher risk for PTSD to implement early intervention. Similarly, we opted to retain one study that focused on PTSD due to subarachnoid hemorrhage, which clinicians and epidemiologists view as a distinct condition from stroke/TIA, because of the similarities in patient experience and because the aggregate estimate was not sensitive to its exclusion. Future research should determine whether PTSD due to subarachnoid hemorrhage is rightly grouped with PTSD due to stroke/TIA.

It is also important to note the difference in prevalence estimates reported in studies that assessed PTSD by screening questionnaire versus clinical interview. Although screening questionnaires are often used to identify patients who should be further assessed for PTSD diagnosis–and therefore may overestimate prevalence–reliance on screening questionnaires for estimates of stroke-induced PTSD prevalence may further inflate those estimates due to overlap in symptoms of stroke and PTSD. Very little is known about the validity of PTSD questionnaire items in stroke survivors, but the evidence that exists suggests that PTSD questionnaires are valid in this population. In one study, confirmatory factor analysis of responses to the PTSD Checklist-specific for stroke conformed to the established factor structure for PTSD due to other types of events [13]. In another small study, PTSD severity was not related to either lesion site or neurologic and memory deficits that could be expected to inflate PTSD symptoms were the scale not valid [6]. Further research on the influence of neurologic and cognitive deficits on PTSD symptom expression is sorely needed. Finally, future research should also consider concomitance with other comorbid stroke factors including depression, however, it is important to note that the effect of ACS-induced PTSD on ACS recurrence and mortality is independent of depression [3].

### Conclusions

Across published studies estimating the prevalence of stroke-induced PTSD, two general conclusions can be drawn: (1) stroke-induced PTSD is relatively common, with approximately one in four experiencing PTSD in the first year after stroke and one in nine experiencing chronic PTSD over a year later, and (2) based on a single study, stroke-induced PTSD symptoms appear to influence important secondary prevention behaviors such as medication adherence [13], independent of depression. Though no data now exist to address the issue, if the risk of stroke or other cardiovascular event recurrence associated with stroke-induced PTSD is comparable to that found for PTSD after ACS, stroke-induced PTSD may be a significant, novel risk factor for recurrent stroke, especially given its high prevalence after stroke. Screening for PTSD in a large, population-based prospective cohort study would yield definitive prevalence and secondary risk estimates, and should be a priority for researchers. In the meantime, clinicians should be mindful that PTSD can be a devastating mental health condition and should consider screening for PTSD in stroke survivors.

## Supporting Information

Checklist S1 **PRISMA checklist.**
(DOC)
